# Direct Arylation Synthesis of Small Molecular Acceptors for Organic Solar Cells

**DOI:** 10.3390/molecules28083515

**Published:** 2023-04-16

**Authors:** Xiaochen Wang, Yuechen Li, Jianfeng Li, Yuan Zhang, Jinjun Shao, Yongfang Li

**Affiliations:** 1 School of Materials Science and Engineering, Shaanxi Normal University, Xi’an 710119, Chinaliyf@iccas.ac.cn (Y.L.); 2 Department of Materials Science and Engineering, Southern University of Science and Technology (SUSTech), No. 1088, Xueyuan Road, Shenzhen 518055, China; 3 Key Laboratory of Flexible Electronics (KLOFE), School of Flexible Electronics (Future Technologies), Nanjing Tech University, Nanjing 211816, China; 4 Beijing National Laboratory for Molecular Sciences, CAS Key Laboratory of Organic Solids, Institute of Chemistry, Chinese Academy of Sciences, Beijing 100190, China

**Keywords:** direct arylation, C-H bond activation, organic solar cells (OSCs), small molecular acceptors (SMAs)

## Abstract

In recent years, small molecular acceptors (SMAs) have extensively promoted the progress of organic solar cells (OSCs). The facile tuning of chemical structures affords SMAs excellent tunability of their absorption and energy levels, and it gives SMA-based OSCs slight energy loss, enabling OSCs to achieve high power conversion efficiencies (e.g., >18%). However, SMAs always suffer complicated chemical structures requiring multiple-step synthesis and cumbersome purification, which is unfavorable to the large-scale production of SMAs and OSC devices for industrialization. Direct arylation coupling reaction via aromatic C-H bonds activation allows for the synthesis of SMAs under mild conditions, and it simultaneously reduces synthetic steps, synthetic difficulty, and toxic by-products. This review provides an overview of the progress of SMA synthesis through direct arylation and summarizes the typical reaction conditions to highlight the field’s challenges. Significantly, the impacts of direct arylation conditions on reaction activity and reaction yield of the different reactants’ structures are discussed and highlighted. This review gives a comprehensive view of preparing SMAs by direct arylation reactions to cause attention to the facile and low-cost synthesis of photovoltaic materials for OSCs.

## 1. Introduction

Organic solar cells (OSCs) have generated significant interest in both research and industrial communities due to their inherent advantages. Compared with other types of solar cells [1,2,3,4], OSCs show the features of tunable optoelectrical properties, high flexibility, lightweight, and facile large-area devices fabrication through low-cost solution-processing techniques [5,6,7]. In recent years, small-molecule acceptors (SMAs) significantly promote the development of OSCs and improve the power conversion efficiency (PCE) of OSCs [8,9,10,11,12]. SMAs have been proven to be the most efficient electron-accepting materials for OSCs. Moreover, SMA-based OSCs have realized PCEs of over 18% [13,14,15,16,17,18,19]. However, the present high-performance SMAs [4,5] with complicated molecular structures usually require multiple-step synthesis and cumbersome purification which results in low synthetic yield, high cost, and difficult large-scale production. Hence, designing efficient SMAs with simple structures and reducing the synthesis steps and complexity become one of the most important topics for the development of OSCs. Nearly all strategies to synthesize high-efficiency SMAs involve Stille-coupling reactions to form aryl-aryl bonds to construct π-conjugated structures. However, the Stille-coupling reaction requires the pre-activation of the reactant by introducing costly organotin moieties through highly reactive organometallic reagents (mainly *n*-butyl lithium) under strictly anhydrous and oxygen-free conditions at low temperatures (e.g., −78 °C). In addition, the process of producing Stille coupling products is accompanied by the formation of stoichiometric quantities of toxic trialkyl-stannanes by-products. Obviously, the excessive synthesis steps at strict conditions, cumbersome purification, and poisonous by-products will lead to severe synthetic difficulty and cost. At the same time, the pre-activation and toxic wastes tend to induce safety and environmental concerns.

In contrast, direct arylation coupling reaction directly uses aryl C-H bonds of (hetero) aryl derivatives as coupling partners to generate aryl-aryl structures [20,21,22,23,24,25], which gets rid of the pre-functionalization of (hetero)arenes. This strategy simplifies the preparation and purification, improves atom economy, and generates only environmental-benign wastes. These advantages are favorable to decreasing the synthetic difficulty and overall cost of π-conjugated molecules. Therefore, the direct arylation coupling reaction benefits the preparation of π-conjugated polymers and small molecules by reducing reaction steps and simplifying the synthetic processes.

Direct arylation coupling has been studied intensively in the past few years. It has been demonstrated as a promising and practical approach in preparing π-conjugated materials for OSCs [26,27,28], which is conducive to the development and application of OSCs. Without concern for structure defect residues, the production of small molecule semiconductors and monomers for π-conjugated polymers should more readily benefit from the convenient and straightforward direct arylation coupling reaction. However, the research and development of the synthesis of SMAs by direct arylation reactions were far inferior to that of conjugated polymer photovoltaic materials [27,29,30,31,32]. The present review aims to summarize the progress of the synthesis of SMAs by direct arylation and highlight the influence of reaction conditions of direct arylation on the reactivity and selectivity of typical building blocks in SMAs.

## 2. Synthesis of Small-Molecule Acceptors

**Figure 1:**
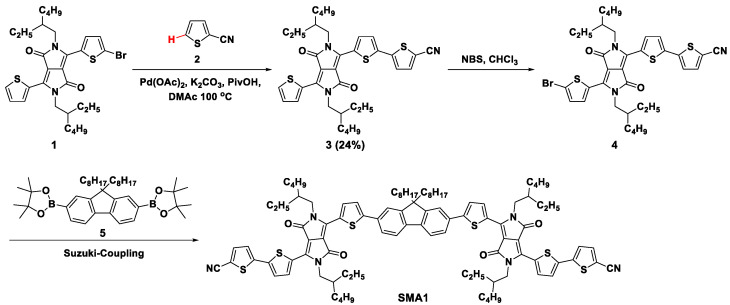
Synthetic route of **SMA1** through Pd(OAc)_2_-catalyzed direct arylation coupling reaction [33].

In 2016, Chen et al. reported the synthesis of acceptors **SMA1** by the direct arylation coupling reaction [33]. As shown in Figure 1, the electron-withdrawing group thiophene-2-carbonitrile (**2**) was connected to mono-brominated thiophene-diketopyrrolopyrrole (DPP) **1** through direct arylation coupling reaction under the catalyst of palladium(II) acetate (Pd(OAc)_2_). However, because of the competition of the self-coupling reaction of **1**, compound **3** was obtained at a low yield of 24%. After bromination, the intermediate compound **4** further reacted with fluorene diboronic ester (**5**) through Suzuki coupling to afford the final acceptor **SMA1** in 70% yield. **SMA1** molecules exhibit a high electron mobility of up to 10^−3^ cm^2^ V^−1^ s^−1^ in bulk heterojunction blends. OSCs employing **SMA1** as an acceptor and poly (3-hexylthiophene) (P3HT) as a donor showed a PCE of 2.37% with an open circuit voltage (*V*_oc_) of 0.97 V, a short circuit current density (*J*_sc_) of 6.25 mA cm^−2^, and a fill factor (FF) of 0.39.

**Figure 2:**
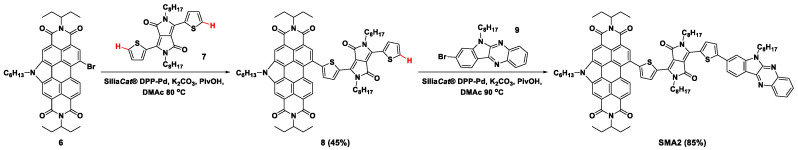
Synthetic route of **SMA2** by Pd-catalyzed two-step direct arylation reaction [34].

**Figure 3:**
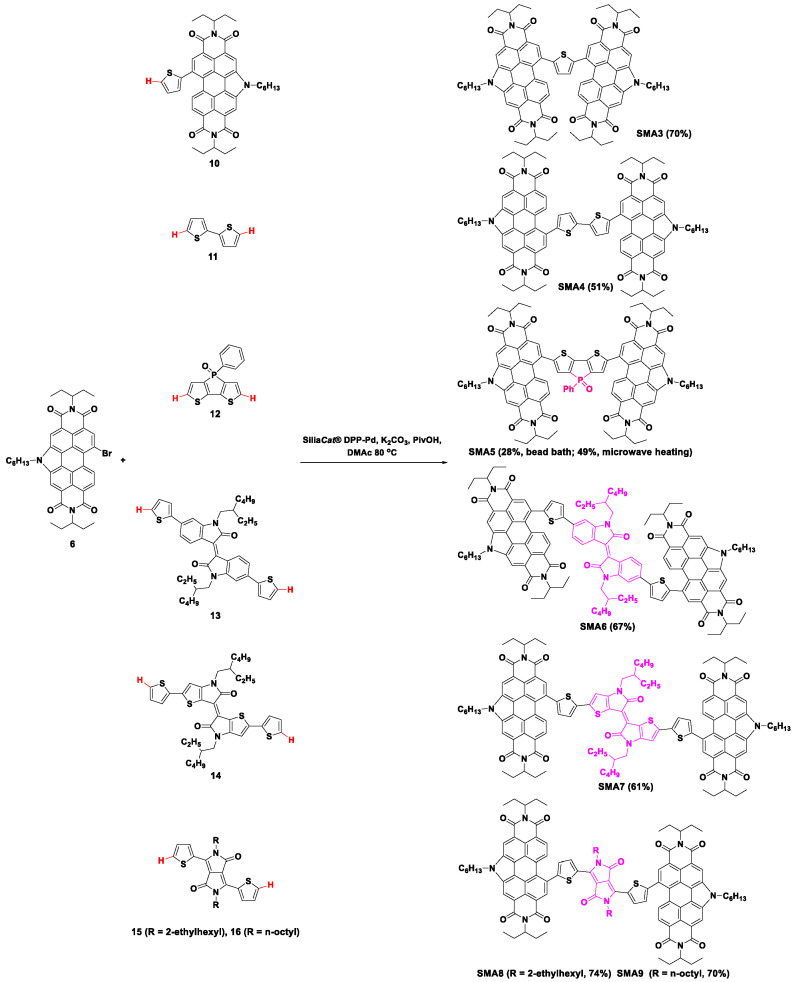
Synthetic routes of **SMA3**–**SMA9** by Pd-catalyzed direct arylation coupling reaction [35,36,37,38,39].

In 2017, an unsymmetrical acceptor **SMA2**, consisting of *N*-annulated perylene diimide (PDI) (**6**), thienyl DPP (**7**), and indoloquinoxaline (**9**) units to enable panchromatic absorption, was synthesized by two-step direct arylation coupling reactions (Figure 2) [34]. In the beginning, **6** reacted with **7** catalyzed with the heterogeneous palladium catalyst *SiliaCat*^®^ DPP-Pd, to obtain the mono-substituted product **8** in 45% yield. Moreover, the following direct arylation coupling reaction was performed between **8** and **9** in the same condition but higher reaction temperature of 90 °C to form the acceptor **SMA2** with a satisfactory yield (85%). However, OSCs taking **SMA2** as the acceptor and P3HT as the donor showed a relatively low PCE of 0.81%. Then, a series of symmetrical acceptors **SMA3**–**SMA9** flanked by *N*-annulated PDI were further prepared [35,36,37,38,39]. Chemical structures and synthetic routes of the acceptors **SMA3**–**SMA9** are shown in Figure 3. PDI monobromide **6** could react with the unactivated thiophene-containing counterparts in the presence of *SiliaCat*^®^ DPP-Pd, potassium carbonate (K_2_CO_3_), and pivalic acid (PivOH) in *N*,*N*-dimethylacetamide (DMAc) to obtain the target products **SMA3**–**SMA9** in moderate yields (51–74%).

Firstly, the thiophene- and bithiophene-bridged compounds **SMA3** and **SMA4** were synthesized by direct heteroarylation [35]. Using a combination of **6** and thiophene-PDI synthon **10**, **SMA3** was synthesized with a yield of 70%. While the bithiophene **11** coupling partner was utilized to react with **6** by double direct arylation coupling to form small molecule **SMA4** in 51% yield. The lowest unoccupied molecular orbital (LUMO) energy levels of the acceptors were relatively high, which was beneficial to obtain the high *V*_oc_ of the corresponding OSCs. As a result, the *V*_oc_ of the OSCs using PTB7-Th as the donor and **SMA3** and **SMA4** as the acceptor reached 0.99 V and 1.05 V, respectively. However, due to the lack of tendency for the acceptors to form crystalline domains, the blend films using **SMA3** and **SMA4** as acceptors and PTB7-Th as donors presented poor morphology, which resulted in low *J*_sc_ and low fill factors for the corresponding OSCs. Finally, the PCE of the OSCs based on PTB7-Th:**SMA3** and PTB7-Th:**SMA4** were only 2.02% and 2.74%, respectively (Table 1).

Bithiophene is one of the most commonly used building blocks in organic semiconductors because of its electronic properties, easy preparation, and high stability. More importantly, the properties of bithiophene-based materials are readily tunable through the functionalization of the building blocks. The novel way to develop bithiophene derivative building blocks is to incorporate a bridge unit at the 3- and 3′-positions to form a fused five- or six-membered ring system or to insert a π-conjugated unit between the two thiophenes to generate a large π-conjugated system. Thus, a bithiophene derivative dithienophosphole oxide (**12**) was exploited as the central core to synthesize the new acceptor **SMA5** (Figure 3) [36]. Owing to the high sensitivity of the phosphole-containing structure under hot and basic conditions, the direct arylation reaction was carefully optimized to restrain the decomposition of the dithienophosphole oxide unit. Employing the optimized conditions (*SiliaCats*^®^ DPP-Pd, K_2_CO_3_, and PivOH in DMAc, 80 °C), **SMA5** was obtained in 28% yield through bead bath and in 49% yield through microwave heating. Compared with that of its phosphole-free analog **SMA4**, the phosphole unit could efficiently lower the energy levels of **SMA5**. As a result, using PTB7-Th as a donor, OSCs based on PTB7-Th:**SMA5** showed a slightly decreased *V*_oc_ (0.99 V) than that of **SMA4** (1.05 V). Combined with the reduced *J*_sc_, the PCE of **SMA5**-based OSCs (2.05%) is slightly lower than that of **SMA4** (2.74%). Nevertheless, all **SMA5**-based devices using different electron donors, including PTB7-Th, PBDB-T, and PDTT-BOBT, presented high *V*_oc_ in the range of approximately 1.0~1.1 V, paving the way for the further design of SMAs for OSCs with high *V*_oc_.

To expand the design strategy of *N*-annulated PDI-flanked acceptors and obtain high photovoltaic performance based on the molecular skeleton of **SMA4**, new acceptor molecules **SMA6**, **SMA7**, and **SMA8**, were prepared by incorporating isoindigo, thienoisoindigo, and DPP in the middle of bithiophene as shown in Figure 3 [37]. Moreover, these acceptors were prepared from *N*-annulated PDI monobromide **6** and bisthiophene-substituted counterparts (**13**, **14**, and **15**) by direct arylation reaction in the presence of *SiliaCat*^®^ DPP-Pd, K_2_CO_3_, and PivOH in DMAc and the target products were isolated in appreciable yields of 67%, 61%, and 74% for **SMA6**, **SMA7**, and **SMA8**, respectively. Because of the nature of the dye core (i.e., isoindigo, thienoisoindigo, and DPP), **SMA6**, **SMA7**, and **SMA8** displayed larger absorption coefficients and additional absorption peaks or shoulders in longer wavelength regions, compared to **SMA4**, which could benefit the absorption and utilization of light. PTB7-Th was used as an electron donor to evaluate the influence of the different dye cores on the photovoltaic performance of these acceptors. PCE reached 2.6%, 0.4%, and 2.9% for the **SMA6**-, **SMA7**-, and **SMA8**- based OSCs, respectively. This result indicated that thiophene-DPP was the superior bridge in this series of *N*-annulated PDI-flanked acceptors. At the same time, the DPP-embedded acceptor **SMA8** was proved to be sufficiently amenable to the *SiliaCats*^®^ DPP-Pd catalyzed direct arylation coupling reaction, and it could be prepared with the highest yield.

The thiophene-DPP unit has been widely applied for organic optoelectronic devices. Moreover, thiophene-DPP **15** was synthesized from its un-alkylated precursor. Hence, it is easy to change the alkyl side chains attached to the nitrogen atoms of the DPP moiety to tune the assembly and aggregation properties of the molecules. Therefore, DPP-based material **SMA9** with linear n-octyl substituents on the DPP core was readily prepared from **16** (Figure 3) [38]. Compared to **SMA8**, **SMA9** exhibited obvious thin-film re-organization upon taking 1,8-diiodooctane as the additive. This re-organization was beneficial for the *J*_sc_ and FF enhancement of **SMA9**-based OSCs to accomplish an improved efficiency of 4.1%. Moreover, it was found that **SMA9** displayed a favorable response to post-deposition solvent vapor annealing (SVA) conditions. The chloroform SVA treatment remarkably changed the absorption profile, film morphology, and charge carrier mobility of the **SMA9**-based active layer, and the consequent photovoltaic performance of the corresponding OSCs. As a result, the PCE of OSCs based on PTB7-Th:**SMA9** reached 5.6%, with a higher *J*_sc_ of 11.32 mA cm^−2^.

To meet the demands of widespread use and large-area OSC devices, a scale-up synthesis of **SMA9** was carried out, and a screening of benzo[1,2-*b*:4,5-*b*′]dithiophene (BDT)-based donor polymers was investigated [39]. Taking advantage of the direct arylation cross-coupling protocol and the re-usable silica-supported palladium catalyst *SiliaCats*^®^ DPP-Pd, **SMA9** was prepared with an isolated yield of 68% at a multi-gram scale. To the best of our knowledge, this was the largest scale-up synthesis of the acceptor materials by direct arylation reaction. The resulting **SMA9** was paired with different BDT-based donor polymers, i.e., PTB7-Th, TTFQx-T1, PBDB-T, and J61, to construct OSCs, and PCE of the OSCs was in the range of 2.3–5.7%, as shown in Table 1. After interfacial modification, the PCE of solar cells based on PTB7-Th:**SMA9** was improved to 6.2%, with a significantly increased *J*_sc_ of 14.52 mA cm^−2^.

**Figure 4:**
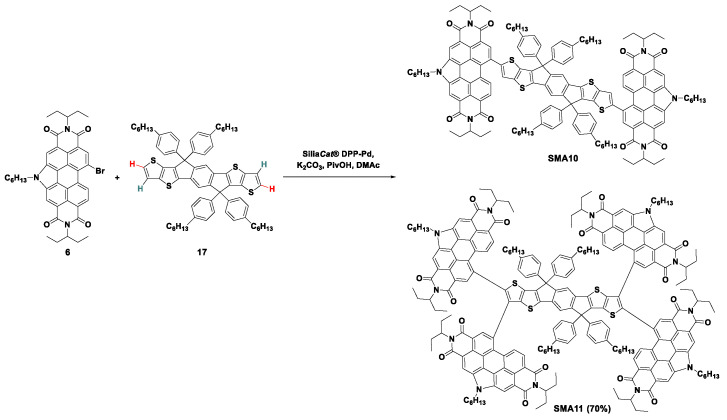
Synthetic route of **SMA11** by *SiliaCats*^®^ DPP-Pd-catalyzed direct arylation coupling reaction [40].

In 2018, the *SiliaCats*^®^ DPP-Pd catalyzed direct arylation reaction was extended to synthesize an indacenodithieno[3,2-*b*]thiophene (IDTT) linked *N*-annulated PDI acceptor [40]. Surprisingly, instead of the bis-substituted product **SMA10**, the *SiliaCats*^®^ DPP-Pd catalyzed reaction proceeded largely to the tetra-substituted product **SMA11** as shown in Figure 4. This result provided a new way to access the quadruple PDI compound. By increasing the feed ratio of **6** and **17** to 4.2 and the reaction temperature to 120 °C, **SMA11** was obtained as a fine dark red crystalline powder in 70% yield. As expected, with four PDI units, **SMA11** displayed a high molar absorption coefficient exceeding 200,000 M^−1^ cm^−1^, approximately four times that of monomeric PDI species. OSCs taking the polymer PTB7-Th as a donor and **SMA11** as an acceptor were fabricated to study the photovoltaic performances. The inverted bulk-heterojunction (BHJ) OSCs based on PTB7-Th:**SMA11** showed a good photovoltaic response with PCEs of 3.4% and a high *V*_oc_ of up to 1.0 V. The hole and electron mobilities of the PTB7-Th:**SMA11**-based active layer were 2.74 × 10^−4^ cm^2^ V^−1^ s^−1^ and 4.00 × 10^−6^ cm^2^ V^−1^ s^−1^, respectively, through space charge-limited current (SCLC) measurement. The active layer’s low electron mobility and unbalanced charge transport should be one of the most important reasons for the low *J*_sc_ (8~10 mA cm^−2^) and FF (0.34~0.35). All in all, this study provided a new way for the synthesis and further development of tetra-substituted IDTT derivatives by C-H direct arylation.

Due to steric hindrance between the *α*- and *β*- substituents, the *N*-annulated PDI units were almost perpendicular to the IDTT core. Thus, there is minimal electron delocalization between the IDTT and PDI units. This contributed to the lack of prolonged wavelength absorption and low electron mobility of **SMA11**. Perhaps the *α*,*α*-bis-substituted PDI-IDTT product **SMA10** with better planarity and conjugation between PDI and IDTT segments should make better use of sunlight and obtain higher electron mobility and improved photovoltaic performance. On the other hand, the formation of this product demonstrated that both *α*-C-H and *β*-C-H bonds in thiophenes of the IDTT core were simultaneously activated under the *SiliaCats*^®^ DPP-Pd catalyzed condition. In fact, the unselective C–H activation was considered the major drawback of direct arylation reactions, which decreased reaction yields in the synthesis of small molecules and caused structural defects in the synthesis of polymers. The *β*-defects (branching defects) severely degraded the photovoltaic properties of the resulting polymers and cannot be removed [41,42]. Besides the limited C-H bond selectivity, the heterogeneous palladium catalyst in strong a polar solvent DMAc also caused solubility issues in the resulting SMAs. As a result, *SiliaCat*^®^ DPP-Pd catalyzed direct arylation mainly generated the coupling products with a yield of not more than 70%.

**Figure 5:**
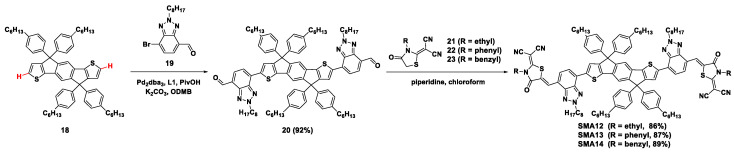
Synthetic routes of **SMA12**–**SMA14** involved Pd_2_dba_3_-catalyzed direct arylation coupling reaction [40].

Moreover, our previous studies showed that nonpolar solvent conditions could offer direct arylation with more excellent selectivity and higher reaction activity [43]. This approach was extended to synthesize an A-D-A trimer involving indacenodithienothiophene (IDT) derivatives to construct SMAs (Figure 5). With the catalyst of tris(dibenzylideneacetone)dipalladium(0) (Pd_2_(dba)_3_)/tris(o-methoxyphenyl)phosphine (P(*o*-MeOPh)_3_, **L1**), in the presence of K_2_CO_3_ and PivOH in 1,2-dimethylbenzene(ODMB), 4-formyl benzotriazole (BTA) bromide **19** was coupled with IDT **18** to achieve the *α*,*α*-bis-(aldehyde benzotriazole) flanked IDT (**20**) in an excellent yield of 92%, with only trace *α*-mono-substituted product. In addition, no *β*-substituted (tris- and tetra-substituted) products were identified [44]. Based on the critical intermediate di-aldehyde **20**, **SMA12**–**SMA14** with different end groups were synthesized by a Knoevenagel condensation reaction. Using J52-F as the electron donor, the PCE reached 9.04%, 5.61%, and 11.27%, for **SMA12-**, **SMA13-**, and **SMA14**-based OSCs, respectively.

**Figure 6:**
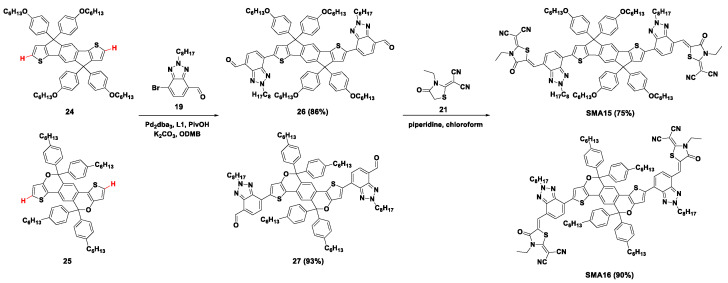
Synthetic routes of **SMA15** and **SMA16** by Pd_2_dba_3_-catalyzed direct arylation coupling reaction [45].

Later, this protocol was further applied to prepare the similar A-D-A type acceptors **SMA15** and **SMA16** with various electron-donating cores, as shown in Figure 6. Through the direct arylation coupling reaction between IDT derivative **24** or its analog **25** and **19,** the intermediates **26** and **27** were obtained in high yields of 86% and 93%, respectively. The dialdehydes were subsequently reacted with dicyanomethylene-3-ethylrhodanine (**21**) in the presence of piperidine to produce **SMA15** and **SMA16**. This type of SMA proved to be a promising candidate for P3HT-based OSCs. Solar cells taking P3HT:**SMA15** and P3HT:**SMA16** as active layers exhibited relatively high PCE of 6.56% and 6.31% [45].

**Figure 7:**
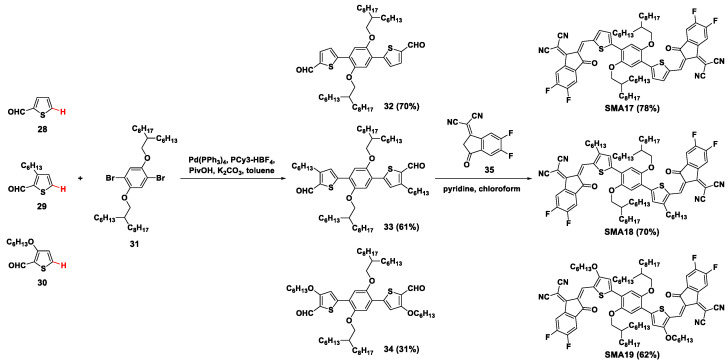
Synthetic routes of **SMA17**–**SMA19** involved Pd(PPh_3_)_4_-catalyzed direct arylation coupling reaction [46].

By direct arylation coupling and Knoevenagel condensation, non-fused ring electron acceptors **SMA17**–**SMA19** were readily prepared (Figure 7) [46]. The thiophene-2-carbaldehydes (**28**–**30**) reacted with 1,4-dibromo-2,5-bis((2-hexyldecyl)oxy) benzene (**31**) with the catalyst of tetrakis(triphenylphosphine)palladium(0) (Pd(PPh_3_)_4_)/tricyclohexylphosphonium tetrafluoroborate (PCy_3_·HBF_4_) to produce the dialdehyde intermediates **32**–**34** in yields of 70%, 61%, and 31%. Then, the target products were accessed from the condensation reaction of the dialdehydes and 5,6-difluoro-1,1-dicyanomethylene-3-indanone (**35**). Employing PBDB-TF (PM6) as an electron donor, the PCE of OSCs taking **SMA17**, **SMA18**, and **SMA19** as the acceptors were 4.08%, 10.27%, and 6.62%, respectively.

**Figure 8:**
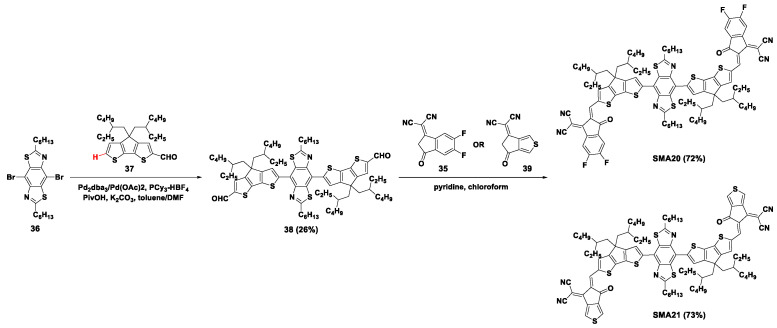
Synthetic routes of **SMA20** and **SMA21** through direct arylation reactions [47].

In the same year, electron-withdrawing building blocks benzobis(thiazole) with quinoid-resonance effect were employed as the core to construct non-fused acceptors **SMA20** and **SMA21**, as shown in Figure 8 [47]. The benzobis(thiazole) dibromide (**36**) was coupled with 4*H*-cyclopenta[2,1-*b*:3,4-*b*′]dithiophene (CPDT) carboxaldehyde (**37**) in a complicated direct arylation reaction condition containing double catalysts (Pd_2_dba_3_ and Pd(OAc)_2_) and double solvents (*N*,*N*-dimethylformamide (DMF) and toluene) in the presence of PCy_3_·HBF_4_ ligand, K_2_CO_3_ base, and PivOH additive. However, this condition only gave the trimer intermediate **38** with a low yield of 26%. The dialdehyde intermediate was treated with **35** or 2-(6-oxo-5,6-dihydro-4*H*-cyclopenta[*c*]thiophen-4-ylidene)malononitrile (**39**) with the catalyst of pyridine to afford the target acceptors **SMA20** and **SMA21**, in yields of 72% and 73%, respectively. Using PBDB-T as the electron donor, **SMA20** and **SMA21**-based OSCs exhibited high PCE of 11.50% and 10.17% with high *J*_sc_ of 21.80 mA cm^−2^ and 17.97 mA cm^−2^, respectively.

**Figure 9:**
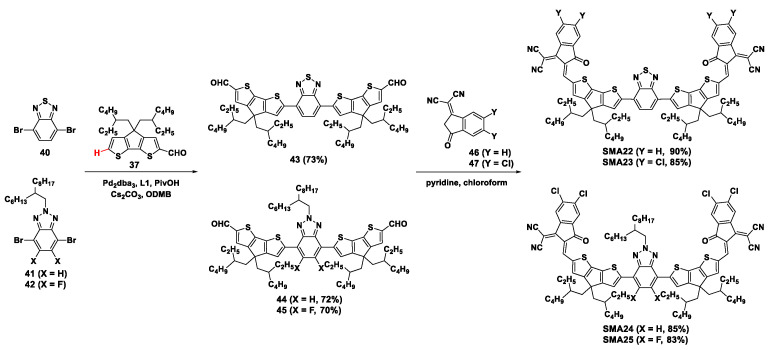
Synthetic routes of **SMA22**–**SMA25** through Pd_2_dba_3_-catalyzed direct arylation coupling reaction [48,49].

Recently, the direct arylation coupling between CPDT carboxaldehyde (**37**) and electron-deficient benzo-2,1,3-thiadiozole (BT) and BTA derivatives (**40**, **41**, and **42**) was carried out to prepare the intermediates of non-fused ring acceptors **SMA22**–**SMA25**, as shown in Figure 9 [48,49]. In the presence of Pd_2_dba_3_/**L1**, PivOH, and cesium carbonate (Cs_2_CO_3_) in ODMB, the dialdehyde intermediates **43**, **44**, and **45** were produced in good yields of 73%, 72%, and 70%, respectively. Then, the dialdehyde compounds were subject to Knoevenagel condensation with 1,1-dicyanomethylene-3-indanone (**46**) and its dichloride (**47**) to obtain the acceptors **SMA22**–**SMA25** in yield of 83% to 90%. The photovoltaic properties of the acceptors were evaluated in solar cells by employing PBDB-T, PBDB-T-2Cl, and PM6 as the electron donors. The PCE of OSCs based on PBDB-T/**SMA22** and PBDB-T-2Cl/**SMA22** was 9.3% and 2.8%, respectively. In contrast, OSCs based on PBDB-T/**SMA23** and PBDB-T-2Cl/**SMA23** achieved high PCE of 10.2% and 10.5%, respectively. Moreover, the OSCs based on PM6/**SMA24** and PM6/**SMA25** exhibited PCE of 8.50% and 10.56%, respectively.

**Figure 10:**
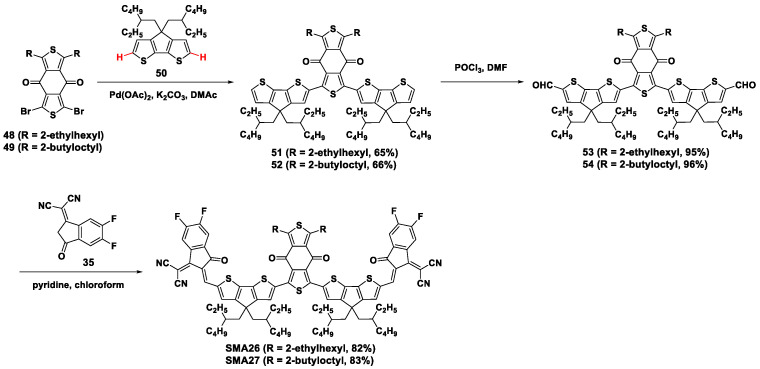
Synthetic routes of **SMA26** and **SMA27** by Pd(OAc)_2_-catalyzed direct arylation reaction [50].

Similar non-fused ring acceptors **SMA26** and **SMA27** were prepared using benzodithiophenedione (BDD) core with 2-ethylhexyl or 2-butyloctyl side chains, respectively (Figure 10) [50]. Firstly, Pd(OAc)_2_-catalyzed direct arylation coupling was performed between BDD dibromides **48** or **49** and CPDT compound **50** to obtain intermediate products **51** and **52** with yields of around 65%. Subsequently, formyl groups were introduced by the *Vilsmeier-Haack* reaction at the *α*-position of the thiophene unit in the trimer intermediates to obtain the aldehyde-functionalized **53** and **54** in high yields. Finally, the target **SMA25** and **SMA26** were produced by *Knoevenagel* condensation with good yields of 82% and 83%, respectively. Using PM6 as the electron donor, the OSCs based on **SMA26** afforded a high PCE of 12.59% with an excellent *J*_sc_ of 22.57 mA cm^−2^, a *V*_oc_ of 0.88 V, and an FF of 63.38%. In comparison, the counterpart devices based on **SMA27** showed a lower PCE of 9.80%, with *J*_sc_ of 19.09 mA cm^−2^, *V*_oc_ of 0.87 V, and FF of 58.36%.

**Figure 11:**
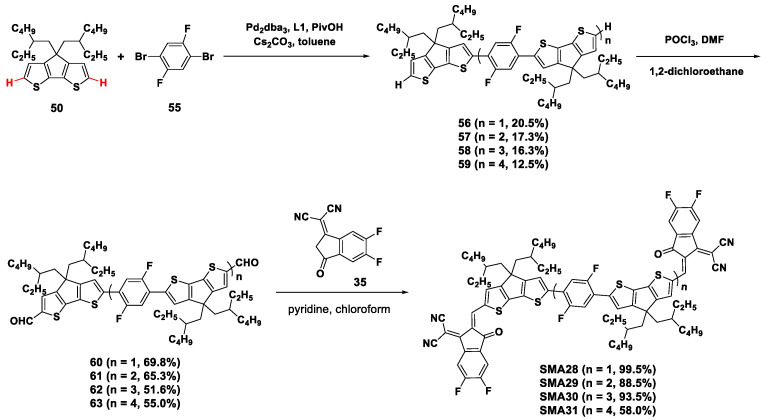
Synthetic routes of **SMA28**–**SMA31** through Pd_2_dba_3_-catalyzed direct arylation reaction [51].

**Figure 12:**
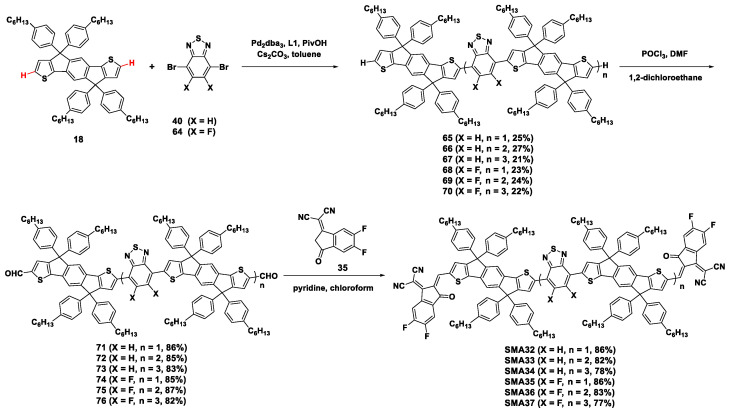
Synthetic routes of **SMA32**–**SMA37** by Pd_2_dba_3_-catalyzed direct arylation reaction [52].

In addition, SMAs consisting of oligomeric cores were designed and synthesized via the one-pot direct arylation reaction, as shown in Figure 11 and Figure 12 [51,52]. CPDT compound **50** reacted with 1,4-dibromo-2,5-difluorobenzene (**55**) in the presence of Pd_2_dba_3_, **L1**, Cs_2_CO_3_, and PivOH in toluene, to obtain the oligomers [51]. With the molar ratio of 1.6:1, **56**–**59** were obtained in the yield of 20.5%, 17.3%, 16.3%, and 12.5%, respectively. Through the *Vilsmeier-Haack* reaction and *Knoevenagel* condensation, the acceptors **SMA28**–**SMA31** were produced in reasonable yields (Figure 11). Subsequently, PBDB-T was used as the donor to evaluate the photovoltaic properties of the acceptors. With an extension of the oligomer linker, PCE of the corresponding OSCs firstly increased from 6.31% (**SMA28**) to 9.32% (**SMA29**), then gradually decreased to 5.71% (**SMA30**) and 2.76% (**SMA31**). Moreover, the *J*_sc_ and FF values of the devices showed the same tendency. While benefiting from the progressively promoted LUMO energy levels, the devices’ *V*_oc_ values ascended from 0.77 V to 0.90 V, along with the growth of the linker lengths.

The oligomers **55**–**57** and **68**–**70,** with stepwise chain lengths increasing, were obtained by one-pot direct arylation coupling between IDT (**18**) and BT dibromide (**40**) or its fluoride analog (**64**) with the molar ratio of 2:1 [52]. After formylation, the dialdehyde intermediates reacted with electron-deficient end unit **35** to produce the target A-Linker-A type acceptors **SMA32**–**SMA37** (Figure 12). The photovoltaic performance of these acceptors was evaluated by the OSCs cooperating with PM6 as the donor to investigate the influence of the π-conjugation length of the oligomeric cores. The PCE of the solar cells was 10.27% and 12.08%, for **SMA32**- and **SMA35**-based OSCs, respectively. Longer linkers sharply decreased the PCE of the acceptors to below 5% (i.e., 1.09%, 0.23%, 4.75%, and 1.43% for **SMA33**, **SMA34**, **SMA36**, and **SMA37**, respectively). This result indicated that an overlong linker weakened the D–A electron interactions and decreased the driving force for charge transfer, thus degrading the photovoltaic property of the corresponding acceptors. Increasing the feed ratio is an effective means of suppressing the formation of a higher molecular weight oligomer to improve the yield of the trimer [29]. Increasing the feed ratio of **18** to **40** (or **64**) to 3:1, the reaction yields of the trimer **65** and **68** were improved to about 60% [52]. With a larger feed ratio of 10:1, the reaction yields of the crude trimers reached 83%. These results demonstrated that the oligomer distributions in this type of direct arylation could be facilely tailored by tuning the feeding ratio to obtain the target products.

## 3. Conclusions and Outlook

Direct arylation coupling reaction has produced significant advances in synthesizing π-conjugated polymers and small molecules over the past two decades. This strategy reduces the synthetic steps and production cost of organic semiconductors and makes the synthesis of π-conjugated organic molecules with mild and environmentally benign conditions. This review summarized the synthesis of SMAs in OSCs via direct arylation coupling reaction. These examples demonstrate that the direct arylation coupling can be utilized to prepare a variety of SMAs through an efficient and convenient method. Thiophene and its analogs and derivatives can be effectively arylated to form aryl-aryl π-conjugation structures to construct SMAs or their intermediates. The reaction activity, selectivity, and consequent reaction yield depend on both reaction conditions and the molecular structure of reactants.

The direct arylation protocols Pd_2_(dba)_3_/P(*o*-MeOPh)_3_ in nonpolar solvents (e.g., toluene or ODMB), have been proven to be efficient and universal in the synthesis of a broad range of SMAs. Moreover, the nonpolar solvents toluene and ODMB have much better compatibility with π-conjugated polymers than strong polar solvents, such as DMAc and DMF, to ensure dissolution of both reactants and products and restrain side reactions to the maximum extent. Until now, the studies on the synthesis of electron acceptors using direct arylation coupling have remained relatively limited. Because of the complicated synthesis process of highly efficient SMAs, there is no report focusing on the preparation of fused ring A-DA’D-A type acceptors by direct arylation coupling. Nonetheless, direct arylation coupling should be a standard replacement for more conventional Stille coupling and Suzuki coupling reactions to reduce the synthetic difficulty and preparation cost of SMAs. Hence, the synthetic strategy towards new and existed SMAs should involve direct arylation coupling to facilitate their synthesis and reduce cost. The development of direct arylation reactions in synthesizing SMAs should be essential in driving the research and application of OSCs.

## Figures and Tables

**Table 1:** Photovoltaic properties of the electron acceptors **SMA3**–**SMA9** synthesized by direct arylation.

Acceptor	Donor	*V*_oc_ (V)	*J*_sc_ (mA cm^−2^)	FF	PCE (%)	Ref.
**SMA3**	PTB7-Th	0.99	5.59	36.7	2.02	[35]
**SMA4**	PTB7-Th	1.05	7.17	36.3	2.74	[35]
**SMA5**	PBDB-T	1.10	3.77	29.8	1.24	[36]
**SMA5**	PDTT-BOBT	1.00	6.44	34.8	2.26	[36]
**SMA5**	PTB7-Th	0.99	5.70	36.1	2.05	[36]
**SMA6**	PTB7-Th	1.03	6.97	36.7	2.6	[37]
**SMA7**	PTB7-Th	0.93	1.09	34.2	0.4	[37]
**SMA8**	PTB7-Th	0.97	5.98	50.3	2.9	[37]
**SMA9**	PTB7-Th	0.97	8.10	52.4	4.1	[37]
**SMA9**	PTB7-Th	0.98	11.32	50.1	5.6	[38]
**SMA9**	TTFQx-T1	1.03	9.78	50.2	5.1	[39]
**SMA9**	PTB7-Th	0.98	13.18	44.5	5.7	[39]
**SMA9**	PBDB-T	0.99	6.27	36.5	2.3	[39]
**SMA9**	J61	0.99	6.66	47.5	3.1	[39]

## Data Availability

Not applicable.
